# Agricultural and innovation policies aimed at mitigating climate change

**DOI:** 10.1007/s11356-023-25663-9

**Published:** 2023-02-04

**Authors:** Rosa Puertas, Luisa Marti, Consuelo Calafat

**Affiliations:** 1 Group of International Economics and Development, Universitat Politècnica de València, Camino de Vera S/N, 46022 Valencia, Spain; 2 Departamento de Economía Y Ciencias Sociales, Universitat Politècnica de València, Valencia, Spain

**Keywords:** Climate change, Agriculture, Innovation efficiency, European countries

## Abstract

The EU supports agricultural policies to help farmers meet the challenges of climate change (CC) by promoting more sustainable and environmentally friendly practices. This study focuses on the European primary sector (agriculture, forestry, and fisheries), productive activities that meet humanity’s basic needs, although this sector does not account for a dominant share of GDP. The analysis uses a panel data sample of 22 European countries for the period 2012–2019, and seeks to answer the following research questions: Is there a direct relationship between agricultural innovation efficiency and the technological advances implemented? What effect do GHG emissions and innovation efficiency have on CC? Which agricultural practices have the greatest effect on the volume of GHG emissions? The results indicate that the European primary sector has registered an average rise in productivity of 4%, mainly driven by technological improvements. This underscores the need for agricultural innovation policies that focus not only on improving aspects related to technology but also on making better use of existing resources. In addition, the econometric models estimated confirm that efficiency levels are the most influential determinants of temperature change, while GHG emissions are primarily explained by their own historical values. Ultimately, research and development is a tool that can be used to curb CC, along with the proper use of land and fertilizers. There is thus a need to foster novel agricultural practices that help reduce emissions while ensuring the efficiency of the sector.

## Introduction

The nearly three decades of climate negotiations have made clear the need to achieve sustainable development through a shared global commitment aimed at halting the ongoing environmental degradation. However, perhaps due to the slow pace of implementation or the high cost of some of the approved measures, there has been no respite in the rise in greenhouse gas (GHG) emissions. The Paris Agreement, signed in 2015 and in force since January 2021, prioritizes the goal of keeping the rise in temperature below 2 °C (UNFCCC 2015). Quéré et al. (2021) conclude in their study that achieving this will require an effort 10 times greater than that seen in recent years.

At present, uneven progress is being made around the world. Unlike other countries such as China and the USA, the perseverance of the European Union (EU) member states and the UK in their efforts to curb environmentally damaging practices allowed them to exceed the target set for 2020 in 2017, reducing GHG emissions by 22% compared to 1990 levels. Recently, under the guidelines of the Paris Agreement and backed by European climate law, the EU has raised this target to 55% by 2030, calling for climate neutrality by 2050 (Council of the EU 2021). No economic sector will be able to dodge this regulation, which will require substantial public and private investment. In this regard, the 2021–2027 multiannual financial framework and Next Generation EU provide for 30% of the budget to be allocated to climate-related projects. Specifically, the agricultural sector (agriculture, forestry, and fisheries, AFF^1^) will receive more than 25 billion euros to implement measures related to the environment and climate change (CC), in an effort to ensure sustainable development.

There is a two-way relationship between CC and agriculture: the latter is an activity that not only contributes to global warming, but is also affected by adverse environmental conditions, which have become very common in recent years (Arcenillas 2021). CC, along with population growth, is accelerating resource scarcity and ecological deterioration, creating a need for significant transformations to achieve sustainable agricultural development (Melvani et al. 2022). At the same time, agriculture and deforestation are responsible for almost 24% of global GHG emissions (IPCC 2014). Innovation policies are thus a fundamental tool for increasing the productivity of the primary sector, while reducing negative environmental impacts (de Jong et al. 2016). A shift is needed in the actions undertaken in order to bring about a transformation, realigning economic objectives to achieve a better social, economic, and environmental balance (Andrade et al. 2020).

Following this line of research, some studies have sought to analyze the relationship between innovation and CC mitigation (Dooley and Roberts 2020; Jamil et al. 2021). Others, such as that by Concu et al. (2020), compare and contrast the positions of farmers, researchers, and workers on issues related to the reduction of GHG emissions and adaptation to CC. The results underscore the desirability of implementing active communication chains in order to convey the benefits of innovation on farms. For their part, Leialohilani and Boer (2020) identify the impact of the EU regulatory framework on innovation in the dairy industry and its consumers, reporting contradictory conclusions. While they confirm that the resulting legal clarity has had a positive influence in terms of food safety and consumer protection, they find evidence that the legislation has had adverse effects on innovation.

Against this backdrop, the proposed research seeks to uncover evidence on the aspects of the agricultural sector that could be improved to curb CC. Specifically, a broad analysis is conducted, addressing issues ranging from the efficiency of the innovation implemented by 22 European countries in the period 2012–2019, to the determinants of GHG emissions, all with a focus on the AFF sector. The aim is to answer the following research questions:

- *Q1*. Is there a direct relationship between innovation efficiency in AFF and the technological advances implemented?

*Q1* will be answered by applying a version of data envelopment analysis (DEA), namely DEA-bootstrap, together with the Malmquist index (MI) to detect possible connections.

- *Q2*. What effect do GHG emissions and innovation efficiency have on CC?
- *Q3*. Which agricultural practices have the greatest effect on the volume of GHG emissions?

*Q2* and *Q3* will be answered by applying the generalized method of moments (GMM) to a panel data sample, separately analyzing each determinant under study.

The proposed research will allow us to fill a gap in the literature regarding the sustainable development of the primary sector, providing evidence of the nexus between agriculture and CC. Specifically, this paper makes the following contributions to the literature: (1) it identifies which European countries have more effectively channelled their research and development (R&D) resources, establishing a pattern to guide future policies that ensure the success of the investments in AFF; (2) it assesses the importance of R&D as a way to curb CC; (3) it estimates the influence that certain agricultural practices may have on global warming; and (4) it analyzes a long period thus yielding robust evidence that can be directly applied by decision-makers in order to tackle the environmentally-damaging effects of AFF.

The paper is structured as follows. The “Literature review” section reviews the literature on the importance of primary sector innovation and actions that could foster sustainable agricultural development. The methods and variables used are presented in the “Methods and materials” section. The results of the research are analyzed in the “Results and discussion” section. Lastly, the conclusions, the contribution of the study and the limitations are summarized in the “Conclusions” section.

## Literature review

### Innovation and climate change in the primary sector

In developed economies, the primary sector lives in the shadow of others such as industry or services, and yet authors such as Loizou et al. (2019) have shown it to be at the root of nations’ growth and development. AFF represented just 1.5% of the EU-28 GVA in 2016, a proportion that has changed little in the last 5 years according to the European Commission. It is an activity aimed at meeting humanity’s basic needs and is closely tied to the rest of the system of production. The effects of CC are currently complicating the work of growing crops, rearing livestock or any type of farming that allows farmers to maintain a certain level of income. The natural evolution of AFF over time calls for the introduction of innovative processes that facilitate its adaptation to new market requirements, while also helping to reduce its environmental impact. These considerations should form the core of the policy action adopted by decision-makers (Akkaya et al. 2021). Innovation is a vital tool for curbing the impact of CC on agriculture (Van Passel et al. 2017) and vice versa, enabling companies to adapt continuously to ensure productivity while respecting the environment.

Specifically, AFF will have to deal with increasingly extreme weather and seasonal changes, with regular floods and frosts followed by droughts and heat waves (Kristiansen et al. 2021). According to information from the FAO, the current situation is as follows^2^: a third of all agricultural land is degraded, about 75% of crop genetic diversity has been lost, 22% of livestock breeds are at risk of extinction, a substantial part of the marine fish stocks have been overexploited, and about 13 million hectares a year are converted to other land uses.

The scientific community has mobilized to address this new scenario, the effects of which are becoming increasingly evident and require innovative new approaches to slow them down. The primary sector requires an innovation policy centred not only on economic growth but also aimed at generating change that benefits society (Hekkert et al. 2020), promoting biodiversity, animal welfare, and a cleaner environment, among other issues (Pigford et al. 2018). For example, Deligios et al. (2019) propose a new way of managing irrigation water to mitigate CC-related problems. Repar et al. (2017) suggest new ideas for introducing environmental sustainability on farms, highlighting the need to differentiate between local and global environmental performance indicators.

However, we should not overlook the importance of innovation efficiency as a means of boosting the capacity to tackle CC. In their recent analysis of the efficiency of technological innovation in agricultural production in the US and Europe, Aldieri et al. (2021) conclude that decision-makers should encourage companies to expand their innovation activities, with an increase in R&D investments. This will help improve productivity and encourage knowledge spillovers.

Furthermore, the transformation of the primary sector is of vital importance if we are to be able to eradicate hunger in the world (Sustainable Development Goal, SDG, 2). This goal is directly linked to the rest of the SDGs, all of which are ultimately aimed at ensuring sustainable development. This research seeks to quantify the link between the efficiency of European agricultural innovation and its potential impact on CC (*Q1* and *Q2*). DEA-bootstrap and the MI are used to calculate the efficiency levels of European innovation policies and productivity gains, respectively. GMM is used to determine the importance of efficiency as a tool to mitigate temperature changes (TC), one of the main manifestations of CC.

### Mitigation of agricultural GHG emissions

Most activities in the system of production generate GHG emissions, which have a marked negative impact on the environment and people’s health. In 2018, environmental pollution directly or indirectly caused more than 8 million deaths—1 in 5 deaths in the world—with eastern North America, Europe, and Southeast Asia being the regions with the highest concentration (Vohra et al. 2021). The upward trend in pollution is one of the main problems to be solved in the fight against global warming (Ghani et al. 2019).

The use of agrochemicals such as fertilizers or pesticides boosts production, but is one of the main sources of pollution, giving rise to notable negative impacts on land, water, and air (Shah et al. 2019). Despite the fact these practices are responsible for a significant volume of GHG emissions, they are increasingly being used to respond to market needs, causing major damage to the biosphere. Expósito and Velasco (2020) analyze the potential of European countries to minimize the use of fertilizers without changing their productive capacity. The results confirm the environmental efficiency of the European agricultural sector, showing it to be capable of improving fertilizer management while maintaining the output of its farms. There is a need for a transition towards the use of mineral fertilizers that will enable the achievement of certain SDGs, such as SDG 2 (sustainable agriculture), or SDG 6, which seeks to ensure the availability and sustainable management of water (Ezbakhe 2018).

In addition, the Paris Agreement highlights the role played by land use in climate action (Andrea 2022). According to the EEA, the climate crisis requires sustainable land and soil management, which would facilitate the production of food in sufficient quantities, as well as adaptation to CC. Under the new EU regulation on land use, land use change and forestry, over the next decade EU member states will have to offset GHG emissions generated by land use. These emissions have been quantified in the literature, specifically those from converting grasslands, savannas, and rainforests (Fargione et al. 2008), or from the drainage of peat land forest for palm oil production (Pastowski et al. 2007).

All this reveals a growing concern about how to maintain optimal levels of environmental health while achieving optimal land use. In this vein, Si et al. (2021), using statistical information from China for the period 1990–2012, analyze the effects of agriculture, forestry, and other land uses on GHG emissions, demonstrating a causal relationship. Göpel et al. (2018) argue that CC mitigation policies should be based on sound information about GHG emission forecasts, making it necessary to determine the changing trend in land use and land cover. Other authors such as Wang et al. (2021) analyze the impact of the zero emissions target in New Zealand, while Sun et al. (2019) assess the effects of CC and agricultural land-use changes on agricultural water consumption in a Chinese irrigation district.

Following this line of research, the proposed empirical analysis aims to provide evidence of the impact that innovation efficiency, fertilizers and different land uses can have on European GHG emissions (*Q3*). By so doing, we can add to the existing literature in this area, revealing the appropriate tools for mitigating CC.

## Methods and materials

The empirical analysis has been carried out using a panel data sample of 22 European countries for the period 2012–2019. The incomplete statistical information on some variables has been a limitation, preventing a full analysis of all European countries. As a result, the study sample has been reduced to 22 countries, considered representative of the agricultural and environmental policies adopted in this continent. To answer the three research questions raised, the DEA method, specifically the DEA-bootstrap and MI versions, and GMM have been used.

### Methods: DEA-bootstrap, MI and GMM

DEA has been widely used in the literature to measure the efficiency of activities such as hospitality (Yu and Chen 2020), transport (Mahmoudi et al. 2020) and energy (Abbas et al. 2022), among others. It has also proved valuable for assessing innovation by different EU countries (Kalapouti et al. 2020), and has been applied to environmental issues and eco-innovation (Puertas and Marti 2021).

It is a non-parametric method that can be used to determine the optimal combination of the input and output variables that characterize a set of decision-making units (DMUs), without having to impose a functional form on the relationship between these variables. All DMUs must be defined using the same inputs and outputs. The DEA results indicate the capacity of the DMUs to maximize outputs with the available inputs (output-oriented model) or to minimize the resources needed to reach the established output level (input-oriented model). In addition, depending on whether or not the increases in outputs and inputs are proportional, the model can be defined under the assumption of constant returns to scale (CRS, Charnes et al. 1978) or variable returns to scale (VRS, Banker et al. 1984). Given the characteristics of the sample, in this study, it has been considered more appropriate to use an output-oriented model with VRS, relying on DEA-bootstrap to correct the bias in the estimates of the efficiency indexes by providing confidence intervals (Simar and Wilson 2000). Furthermore, to prevent isolated events from distorting the results, an intertemporal analysis is conducted, covering the period 2012–2019 (Bresciani et al. 2021). The efficiency score ranges between 0 and 1, where 1 corresponds to efficient DMUs. Since the model is output oriented, the level of efficiency is always greater than or equal to 1, with the amount over 1 indicating how much output could be increased using the available resources.

The sequential MI determines the possible productivity changes of DMUs in consecutive periods, incorporating the process of technology accumulation over time (Tulkens and Eeckaut 1995). The MI can be decomposed into a change in the levels of technical efficiency (Efficiency change, EC), and a shift forward in the technological frontier (Technical change or innovation, TecC). If the MI value is greater than 1, it can be said that the DMU in question has achieved improvements in its total factor productivity in the analyzed period. The calculations of efficiency have been done using the statistical package deaR, implemented in Rstudio (Coll-Serrano et al. 2018).

Panel data analyses can be performed using static (individual fixed and random effects) or dynamic models. The latter deal with the main drawback of the former by appropriately addressing endogeneity, taking into account the changing configuration of dependence on the past (Dosi 1988). In short, the dynamic panel models incorporate retroactive effects through instrumental variables, while also accounting for the causal relationships generated within the model. This study employs an extension of the original model presented by Arellano and Bond (1991) and further developed by Roodman (2006), which allows endogenous variables to be instrumented through equations with variables in levels (with lags of increases in variables as instruments) and differences (with lags in levels of the variables as instruments). Specifically, xtabond2 in STATA has been used. This is a method that has been very well received in the scientific community. Its main advantage lies in the fact that it relaxes the requirement for the “strict” exogeneity of inputs; they only have to be predetermined variables (Okoyeuzu et al. 2021; Yao et al. 2022). Thus, four models have been built with the following specifications:

1 $$\begin{array}{cc}\mathrm{Model}\;1&{\;TC}_{it}=\beta_0+\beta_1{{TC}_{it-1}+\beta_2TC_{it-2}+\beta_3EFF}_{it-1}+\beta_4{GHG}_{it}+\varepsilon_{it}\end{array}$$

$${\beta }_{1}, { \beta }_{2},{ \beta }_{3}, {\beta }_{4} >0$$

*i* = 1,….0.22 European countries and *t* = 2012,…… 2019.where *TC* represents the annual temperature change; *EFF* is the level of efficiency; and *GHG* is the emissions. All of this refers to each of the 22 European countries (*i*) that make up the sample for each year analyzed (*t*).

2 $$\begin{array}{cc}\mathrm{Model}\;2&{GHG}_{it}=\beta_0+\beta_1{{GHG}_{it-1}+\beta_2EFF}_{it-1}+\beta_j{\mathrm{Fertilizers}}_{jit}+\varepsilon_{it}\end{array}\;$$

$${\beta }_{1}, {{ \beta }_{2}, \beta }_{j}>0$$

*i* = 1,….0.22 European countries, *t* = 2012,……2019, and *j* = nitrogen, phosphorus, and potassium

3 $$\begin{array}{cc}Model\;3&{GHG}_{it}=\beta_0+\beta_1{{GHG}_{it-1}+\beta_2EFF}_{it-1}+\beta_j\mathrm{Share}\;\mathrm{in}\;\mathrm{land}\;{\mathrm{area}}_{jit}+\varepsilon_{it}\end{array}\;$$

*i* = 1,….0.22 European countries, *t* = 2012,……2019, and *j* = agricultural land or forest land or cropland divided by land area

$${\beta }_{1},{ \beta }_{2}, {\beta }_{j}>0$$

4 $$\begin{array}{cc}\mathrm{Model}\;4&{GHG}_{it}=\beta_0+\beta_1{{GHG}_{it-1}+\beta_2EFF}_{it-1}+\beta_j\mathrm{Share}\;\mathrm{in}\;\mathrm{agricultural}\;{\mathrm{land}}_{jit}+\varepsilon_{it}\end{array}\;$$

*i* = 1,….0.22 European countries, *t* = 2012,……2019, and *j* = agricultural area under organic agriculture or cropland divided by agricultural land

$${\beta }_{1}, { \beta }_{2}>0; {\beta }_{j} \mathrm{ambiguous}$$

where *GHG* and *EFF* are emissions and efficiency levels, respectively; Fertilizers represents the use of fertilizer by surface area *j* in country *i* in year *t*; Share in land area represents agricultural, cropland, and forest land (*j*) as a share of the total area of country *i* in year *t*; Share in agricultural land represents cropland and the agricultural area under organic farming (*j*) as a proportion of the total agricultural area of country *i* in year *t.*

### Materials

In order to calculate the efficiency levels, a production function defined by inputs and outputs must be constructed, such that the DMUs that achieve an optimal combination of these variables will determine the efficient frontier. Table 1 defines the variables used in the research, all of which refer to AFF and have been sourced from the Eurostat database. Private and public spending on R&D reveals countries’ commitment to agricultural innovation. On the other hand, the number of workers and the GVA indicate the importance of the sector to the national economy, as well as its intrinsic characteristics.

**Table 1:** Variables used in DEA-bootstrap and MI analysis

Variable	Role	Unit	Literature
Government budget allocations for R&D expenditure (GBARD)	Input	Million euro	Guo et al. (2020); Carradedo and Puertas (2021)
Business enterprise R&D expenditure (BERD)	Input	Million euro	Liu et al. (2020); Guo et al. (2021)
Employment (EMP)*	Input	Thousand	Wang et al. (2018); Guo et al. (2021)
GVA at basic prices (GVA)	Output	Million euro	Grovermann et al. (2019); Guo et al. (2021)

*From 15 to 64 years old.

The output-oriented production frontier allows us to determine whether the resources used by the countries have enabled them to maximize the economic activity of AFF. Furthermore, the inputs are lagged by 1 year to take into account the timeframe involved in innovation processes. Table 2 shows the main descriptive statistics of the variables used.

**Table 2:** Descriptive statistics for inputs and outputs (2012–2019)

	GBARD_t-1_	BERD_t-1_	EMP_t-1_	GVA
Mean	140.9	26.5	617.2	11,349.3
SD	199.7	49.7	1099.1	13,254.7
Max	916.7	214.0	5372.8	53,370.2
Min	0.3	0.1	1.6	77.7

The results show a high degree of dispersion in the sample, which is less due to the relative importance of AFF in each economy and more to the different size of the analyzed countries and their commitment to innovation policies. The public sector shows greater involvement than the private sector, albeit with some correspondence between them. Germany, Spain, and France hold the top positions in public and private spending on R&D in AFF. Regarding employment, it should be noted that Malta, Iceland, Belgium, and Norway are the economies whose primary sector absorbs the fewest workers, with Malta, Iceland, Lithuania, and Bulgaria being the countries whose AFF generates the lowest GVA. Table 3 shows the dependent and independent variables that define each of the models. These models assess the link between innovation and CC, as well as other agricultural practices which will help to guide future actions.

**Table 3:** Description of the variables corresponding to each model

Variables	Definition	Source	Unit
Model 1: innovation and GHG versus temperature change			
TC	Temperature change	FAO	°C
GHG	Air pollution (CO_2_, N_2_O, CH_4_, HFC, PFC, SF6, NF_3_) corresponding to AFF	Eurostat	Thousand tonnes
EFF	Level of efficiency	DEA-bootstrap	Values equal to or greater than 1
Model 2: innovation and fertilizer indicators versus GHG emissions			
GHG	Air pollution (CO_2_, N_2_O, CH_4_, HFC, PFC, SF_6_, NF_3_) corresponding to AFF	Eurostat	Thousand tonnes
EFF	Level of efficiency	DEA-bootstrap	Values equal to or greater than 1
Fertilizer indicators	Ratio between the total agricultural use of chemical or mineral fertilizers (nitrogen, phosphorus and potassium) by nutrient and the area of cropland	FAO	Kg/ha
Model 3: land area			
GHG	Air pollution (CO_2_, N_2_O, CH_4_, HFC, PFC, SF_6_, NF_3_) corresponding to AFF	Eurostat	Thousand tonnes
EFF	Level of efficiency	DEA-bootstrap	Values equal to or greater than 1
Share in land area	Provides information on agricultural land, cropland and forest land as a share of the total area of land of each country	FAO	%
Model 4: agricultural area			
GHG	Air pollution (CO_2_, N_2_O, CH_4_, HFC, PFC, SF6, NF_3_) corresponding to AFF	Eurostat	Thousand tonnes
EFF	Level of efficiency	DEA-bootstrap	Values equal to or greater than 1
Share in agricultural land area	Provides information on cropland and organic agriculture as a share of the agricultural area of each country	FAO	%

The variables have been log transformed to prevent the different units of measurement from affecting the results. The dependent variable has been lagged in order to assess the impact of the trend on its value. Table 4 shows the main descriptive statistics for all of them except EFF, as it first needs to be calculated.

**Table 4:** Descriptive statistics and correlation coefficient of the variables for the panel data samples (2012–2019)

		Mean	SD	Max	Min	1	2	3	4	5	6	7	8	9	10
1	TC	1.6	0.6	2.7	0.4	1									
2	GHG	20,013	21,938	76,527	75.3	− 0.06	1								
3	Nitrogen/C	99.9	54.1	399.6	31.4	− 0.05	0.01	1							
4	Phosphate/C	21.3	8.6	73.3	6.2	− 0.14	0.21	0.00	1						
5	Potash/C	24.6	16.8	89.3	3.3	− 0.04	0.04	0.43	0.45	1					
6	AL/LA	43.4	16.5	72.4	2.7	− 0.03	0.41	− 0.03	− 0.01	− 0.09	1				
7	FL/LA	29.2	13.5	61.5	1.2	0.15	0.23	− 0.02	− 0.28	− 0.17	0.81	1			
8	C/LA	30.1	14.8	73.7	0.5	0.30	0.00	− 0.41	− 0.27	− 0.19	− 0.32	− 0.22	1		
9	AAO/AL	6.5	5.0	25.3	0.1	0.34	− 0.13	− 0.16	− 0.19	− 0.07	− 0.15	− 0.09	0.57	1	
10	C/AL	67.8	20.3	100.0	6.5	0.28	− 0.09	0.11	− 0.60	− 0.14	− 0.07	0.43	0.33	0.09	1

*AL* agricultural land, *LA* land area, *FL* forest land, *C* cropland, *AAO* agricultural area under organic agriculture.

The variables show a high degree of variability; in the case of GHG emissions, it exceeds the average value for the analyzed period. France, Germany, and Turkey are the countries whose primary sectors emit the highest volume of GHG, but this does not correspond to a notable use of fertilizers, as is the case with land use. The correlation coefficient shown in Table 4 confirms the independence of the variables analyzed.

## Results and discussion

- *Q1*. Is there a direct relationship between innovation efficiency in AFF and the technological advances implemented?

The intertemporal DEA-bootstrap has been used to calculate the efficiency of public and private innovation policies implemented by a group of significant European countries over an 8-year period. The sequential MI has allowed us to measure possible productivity increases, as well as their source (TecC and EC). The second, third, and fourth columns of Table 5 show the score (EFF score), the level (EFF level), and the number of times that the country in question has been completely efficient (N° EFF), respectively. The EFF score determines the position of each country with respect to the frontier, and the amount over an EFF level of 1 represents how much each country could increase its output (GVA) with the available inputs (R&D expenditure and labor). For example, while Italy could increase it by a little more than 11%, Romania would have to register an increase of 166% to achieve the maximum efficiency score.

**Table 5:** Intertemporal efficiency with DEA-bootstrap and MI (2012–2019)

Countries	EFF score	EFF level	Nº EFF	MI	TecC	EC
Italy	0.901	1.112	4	1.004	1.021	0.984
Norway	0.866	1.162	2	1.074	1.067	1.007
France	0.859	1.167	2	1.036	1.138	0.911
Iceland	0.858	1.170	4	1.104	1.104	1.000
Turkey	0.814	1.238	0	1.011	1.043	0.969
Slovakia	0.812	1.271	1	1.103	1.060	1.041
Finland	0.811	1.247	2	1.063	1.038	1.024
Greece	0.805	1.249	0	0.993	1.031	0.963
Spain	0.796	1.268	2	1.018	1.080	0.943
Lithuania	0.774	1.305	1	1.197	1.196	1.001
Denmark	0.765	1.356	1	1.029	1.047	0.983
Hungary	0.761	1.355	1	0.917	1.102	0.832
Portugal	0.758	1.357	0	1.031	1.071	0.963
United Kingdom	0.740	1.366	1	1.002	1.023	0.979
Germany	0.732	1.378	0	1.031	1.075	0.959
Belgium	0.684	1.474	0	1.033	1.105	0.935
Malta	0.684	1.512	3	1.132	1.163	0.973
Austria	0.672	1.501	0	0.953	1.055	0.903
Czechia	0.596	1.682	0	1.018	1.072	0.950
Bulgaria	0.561	1.801	0	1.058	1.083	0.978
Poland	0.474	2.137	0	1.015	1.037	0.978
Romania	0.385	2.669	0	1.058	1.053	1.005
Mean	0.732	1.444		1.040	1.076	0.967

The results of the MI and the corresponding TecC and EC components are detailed in the last three columns of Table 5. The choice of the sequential MI was motivated by the need to avoid a reversal in TC, which would make little sense in economic terms, as technological advances accumulate over time. On average, it can be seen that the European countries’ primary sectors have experienced productivity increases of 4%; only Greece, Hungary, and Austria have not been able to manage their resources appropriately, registering productivity losses of 0.7%, 8.3%, and 4.7%, respectively. These changes have been mainly driven by improvements in technology (TecC = 1.076), given that efficiency (EC) has registered an average reversal of 3.3%.

The results confirm the absence of a direct relationship between the efficiency score and the advances in productivity (*Q1*). For example, Italy, Norway, France, and Iceland hold the top positions in terms of efficiency levels (they only need to increase their output by 11.2%, 16.2%, and 16.7%, respectively); however, Lithuania (1.197), Malta (1.132), and Iceland (1.104) lead in terms of the MI.

The innovation policies implemented by European countries have resulted in the introduction of technological advances (TecC), which are vital for the primary sector to achieve sustainable development and fulfil the SDGs. In this respect, countries such as Lithuania, Malta, France, Hungary, and Belgium have achieved technological advances of over 10% in the period 2012–2019; however, their efficiency levels show a lot of room for improvement. In this regard, Friha et al. (2021) affirm that R&D must provide solutions to improve not only the productivity but also the efficiency of the agricultural sector. These advances will be reflected in higher production quality and profitability (Farooq et al. 2019).

There are growing calls in the literature for co-innovation in AFF in order to harness synergies, which would improve the efficiency of the outcomes obtained (Fieldsend et al. 2020). Innovation cannot be limited to isolated actions, but rather should be the result of cooperation and interaction between farmers, researchers and anyone in an intermediate position (Lundvall 2016). Pigford et al. (2018) go further still, arguing that it is essential for decision-makers to foster transboundary innovation niches in agricultural systems to support the sustainability of the planet. This will require the design of a systems architecture that facilitates the transition (Meynard et al. 2017).

In Europe, innovation in agriculture has undergone a major transformation, moving away from the linear transmission of knowledge from the researcher to the farmer, towards a more modern, network-like system (Klerkx et al. 2009). Vollaro (2020) reveals a change in the European investment pattern, orchestrated by the Common Agricultural Policy (CAP), bringing about a shift from the sole objective of increasing productivity towards a public commitment to improving environmental sustainability. Furthermore, the European Innovation Partnership for Agricultural Productivity and Sustainability has been developed in an effort to support innovation in the European primary sector, by means of the synergies between the actors involved, as well as alliances between research and practice (Cronin et al. 2022). However, decision-makers must undertake thorough monitoring of the outcomes of the subsidies granted to the sector. Guth et al. (2022), in a comparative analysis of EU and non-EU countries, demonstrate that the environmental outcomes and the technical efficiency of farms do not depend solely on the amount of resources received, thus pointing to the importance of the appropriate use of those resources.

- *Q2*. What effect do GHG emissions and innovation efficiency have on CC?

Global warming has harmful consequences for all humanity, resulting in melting glaciers, changes in the water, and even food shortages. Rising temperatures are considered a consequence of CC, with AFF contributing to the increase, thus necessitating new practices to lessen the impact.

Model 1 has been estimated using GMM. The coefficients have been standardized to determine the relative weight of each of the analyzed variables in terms of TecC (Table 6). All the tests applied confirm the adequacy of the results: the Hansen test confirms that the instruments used are valid and there is no overidentification problem (Prob > chi2 is greater than 0.05); the Arellano-Bond test confirms the absence of second-order serial correlation in the error (AR(2)) (Prob > z is greater than 0.05); the number of instruments is smaller than the number of groups (20 instruments and 22 groups); and the Wald test, with a Prob > chi2 of less than 0.05, indicates that they are correctly specified and the set of indicators explain the dependent variable.

**Table 6:** Two-step GMM estimation results (Model 1)

	Model 1
logTC (-1)	0.0143^**^
logTC (-2)	0.0148^***^
logGHG	0.0233^***^
logEFF (-1)	0.0385^***^
Hansen chi2 (Prob > chi2)	16.79 (0.331)
AR(1) z (Prob > z)	− 2.35 (0.019)
AR(2) z (Prob > z)	0.37 (0.712)
Observations/groups	132/22
Instruments	20

***p < 0.01, **p < 0.05. Hansen, A-Bond, and Wald tests report *p*-values in parentheses.

The results indicate that the efficiency of AFF innovation carried out in the preceding period is the component that has the greatest impact on TC (0.0385). Higher EFF values represent a higher level of inefficiency; therefore, the positive sign confirms its direct relationship with TC; that is, innovation efficiency reduces TC. Second, in terms of importance is the primary sector’s GHG emissions (0.0233), showing that all public actions aimed at reducing emissions entail improvements in TC and CC. Finally, the effect of the trend in TC has also been significant and positive in the two lags analyzed (0.0143 and 0.0148, respectively), yielding evidence that TC has historical memory; that is, high levels in the past have an effect in the present.

The relative importance of AFF in the world economy need not determine private and public resources for R&D in this sector. The efficiency of these investments should not be assessed from an exclusively financial perspective; they also represent a valuable tool for curbing CC. Cotte and Pardo (2021) highlight the importance of innovation as a way to prevent and mitigate the consequences of CC. Smallholder farmers need a policy shift to ensure better access to the resources they need to adapt their farms to be environmentally friendly (Verburg et al. 2019). Specifically, Coderoni and Esposti (2018) have shown that the CAP budget is a key factor in reducing GHG emissions on farms and therefore TC.

These days, in accordance with international agreements on CC, the objective of agriculture is not limited to ensuring food production; it must also be carried out in a way that respects the environment. Vetter et al. (2017) find evidence of the need to modify dietary patterns, encouraging the consumption of cereals, fruits, and vegetables over foods such as meat or rice, which are responsible for the highest volume of emissions. It is therefore vitally important to develop strategies and instruments that reduce agricultural GHG emissions (Jantke et al. 2020). Decision-makers should target aid at the most polluting products (Laborde et al. 2021). Chojnacka et al. (2021) propose the use of local sources of proteins, thus reducing emissions associated with transport. Any actions, no matter how small, focused on achieving this goal will help ensure environmentally beneficial changes over the long term.

- *Q3*. Which agricultural practices have the greatest effect on the volume of GHG emissions?

Three models have been estimated using GMM to assess the impact that certain primary sector practices have on its emissions. The statistical tests applied to each confirm that the conditions have been met to ensure the reliability of the results (Table 7). Again, the coefficients of the variables have been standardized to allow comparison.

**Table 7:** Two-step GMM estimation results (Models 2, 3, and 4)

	Model 2	Model 3	Model 4
logGHG (-1)	0.6692^***^	0.6460^***^	0.6746^***^
logEFF(-1)	0.0019^***^	0.0031^***^	0.0026^***^
logNitrogen/C	0.0015^***^		
logPhosphorus/C	0.0054^***^		
logPotassium/C	− 0.0048^***^		
logAL/LA		0.0214^**^	
logFL/LA		− 0.0158^**^	
logC/LA		0.0241^**^	
logAAO/AL			− 0.0021^**^
logC/AL			0.0015^***^
Hansen chi2(Prob > chi2)	15.58 (0.211)	10.60 (0.225)	15.45 (0.348)
AR(1) z(Prob > z)	− 2.75 (0.006)	− 3.14 (0.002)	− 3.04 (0.002)
AR(2) z(Prob > z)	− 0.83 (0.407)	− 1.08 (0.281)	− 0.91 (0.361)
Observations/groups	154/22	154/22	154/22
Instruments	18	14	18

***p < 0.01, **p < 0.05. Hansen, A-Bond and Wald tests report *p*-values in parentheses.

In all three models, it can be seen that the volume of emissions from the preceding period is the most important factor for current GHG, while innovation efficiency is less so. Model 2 analyzes the impact of fertilizers, considered necessary for plants to grow and produce fruit. Each one has a different function: while nitrogen stimulates plant growth and regeneration, phosphorus is vital for energy transfer and potassium for water absorption. The application of fertilizers increases GHG emissions, and the excessive use of nitrogen and phosphorus fertilizers reduces biodiversity due to contamination of surface water and groundwater. The results confirm their positive relationship with emissions, whereas potassium has a negative relationship. Excess potassium basically damages the seed during germination, reducing the quality of the crop. Therefore, we argue that its sign and its significance do not represent conclusive results. Walling and Vaneeckhaute (2020) demonstrate the existence of a knowledge gap regarding emission factors for potassium fertilizers, recommending a case-by-case study of these factors. For their part, Sikora et al. (2020) recommend the use of slow-release fertilizers as a way to reduce agricultural emissions. Yang et al. (2022) show that higher farmer education with low nitrogen input contribute to eco-efficiency. Along the same lines, Shahbaz et al. (2022) reveal that farmers in a province of Pakistan are changing their use of fertilizers in order to counteract the effects of CC, and also demonstrate the importance of ensuring the efficiency of innovation.

Models 3 and 4 analyze land use, differing in terms of whether they refer to land area or agricultural land, respectively. The resulting estimates confirm that agricultural land and cropland increase GHG emissions, while practices such as forest land and organic farming help mitigate emissions. The study by Skinner et al. (2019) confirms that organic farming reduces GHG in the agricultural sector, as is the case with increasing forest land. Land use in AFF plays an important role in slowing CC, although the measures adopted can adversely affect food security. According to Stevanović et al. (2017), incentive-based policies, such as the protection of carbon-rich forests, should be combined with others that encourage reduced consumption of animal products. Chandio et al. (2022) hold that flexible financial and agricultural policies aimed at the adoption of sustainable practices will translate into benefits for farmers.

## Conclusions

The two-way relationship between CC and AFF calls for careful study. It is only logical that environmental changes have a major influence on the world’s flora and fauna, potentially jeopardizing crop yields and animal reproduction, as well as affecting water resources. This research has sought to provide evidence of the impact that the primary sector has on environmental deterioration, by applying statistical techniques widely used in the literature, namely, DEA-bootstrap, MI, and GMM. The analysis has focused on European countries, which over the past decade have consistently shown their concern about efforts to achieve sustainable development. The study also reveals the need to allocate resources to innovation in the primary sector as a way to halt the environmental deterioration of the planet, while also monitoring their proper application. In addition, upcoming international discussions on CC should address the effect of trends in TC and GHG, as well as the use of chemical fertilizers, or excessive land use for crop cultivation and livestock.

The transition towards an environmentally sustainable society requires a rapid, ongoing transformation of the primary sector to enable it to cope with the consequences of CC. In the short term, minimum tillage techniques, land use planning and organic farming should be encouraged. However, over a longer time horizon, governments need to design economically sustainable agricultural policies that incentivize innovation processes in AFF to ensure that its future development is not compromised. These involve measures to eliminate the trend factor of TC and GHG emissions, which are mainly responsible for the current levels of pollution by AFF. These conclusions are in line with the legislative proposal on sustainable food systems announced by the European Commission for 2023. Specifically, Farm to Fork, the central axis of the European Green Deal, sets the goal of achieving fair, healthy, and environmentally friendly food systems, which requires the appropriate combination of innovation and citizen awareness (European Commission 2020). These strategies should be framed within a set of policies and incentives for agriculture, and accompanied by systems for measuring and assessing results in order to prevent any divergence from the fundamental objective, namely, ensuring the efficiency and environmental sustainability of the sector. CC is a global problem affecting all countries and all economic sectors without exception; hence, the more advantaged nations must help developing countries. What is needed is an integrated communication system where international decision-makers share the technological advances developed for this purpose, while farmers are encouraged to report on the progress achieved.

Despite the long time span analyzed, the main limitation of this research lies in the need to update the results with new statistical information in order to be able to examine the progress made. CC is a global problem and the need to slow it down is becoming ever more pressing; hence, there is a major international commitment to moving forward on instruments that foster sustainable development at all levels—social, economic, and environmental.

## Data Availability

The datasets used and analyzed during the current study are available from the corresponding author on reasonable request.

## Footnotes

1 AFF encompasses all activities dedicated to growing crops, rearing livestock, logging, fishing, and hunting other animals in natural habitats.

2 http://www.fao.org/sustainable-development-goals/overview/fao-and-post-2015/sustainable-agriculture/es/
